# How do clients with multiple problems and (in)formal caretakers coproduce integrated care and support? A longitudinal study on integrated care trajectories of clients with multiple problems

**DOI:** 10.1111/hex.13653

**Published:** 2022-12-15

**Authors:** Lieke Reinhoudt‐den Boer, Jeroen van Wijngaarden, Robbert Huijsman

**Affiliations:** ^1^ Department of Health Services Management & Organisation Erasmus School of Health Policy and Management Rotterdam The Netherlands

**Keywords:** care trajectory, clients with multiple problems (CWMPs), coproduction, integrated care, iterative processes

## Abstract

**Introduction:**

Integrated care can create several advantages, such as better quality of care and better outcomes. These advantages apply especially to clients with multiple problems (CWMPs) who have multiple, interconnected needs that span health and social issues and require different health care (e.g., mental health care or addiction care), social care (e.g., social benefits) and welfare services at the same time. Integrated care is most often studied as a phenomenon taking place at the system, organizational, professional and clinical levels. Therefore, in many studies, clients seem to be implicitly conceptualized as passive recipients of care. Less research has been conducted on how clients and (in)formal caretakers coproduce integrated care.

**Methods:**

We performed a longitudinal study to investigate how CWPMs and (in)formal caretakers coproduce integrated care. Data were collected among CWMPs and their (in)formal caretakers in Rotterdam, the Netherlands. CWMPs' care trajectories were followed for 1–1.5 years. CWMPs were interviewed three times at an interval of 6 months (T0, T1, T2). Informal caretakers were interviewed three times (T0, T1, T2), and formal caretakers of 16 clients were interviewed twice (T1, T2). Data in the municipal record systems about participating CWMPs were also included.

**Results:**

Our study shows that the CWMPs' multidimensional needs, which should function as the organizing principle of integrated care, are rarely completely assessed at the start (first 6 weeks) of CWMPs' care trajectories. Important drivers behind this shortcoming are the urgent problems CWMPs enter the support trajectory with, their lack of trust in ‘the government’ and the complexity of their situations. We subsequently found two distinct types of cases. The highest level of integrated care is achieved when formal caretakers initiate an iterative process in which the CWMP's multidimensional needs are constantly further mapped out and interventions are attuned to this new information.

**Conclusions:**

Our study indicates that integrated care is the joint product of formal caretakers and CWMPs. Integrated care however does not come naturally when CWMPs are ‘put at the center’. Professionals need to play a leading role in engaging CWMPs to coproduce integrated care.

**Patient Contribution:**

CWMPs and their (in)formal caretakers participated in this study via interviews and contributed with their experiences of the process.

## 1 INTRODUCTION

Integrated care has the potential to generate several advantages, including better quality of care (experienced by the client), better continuity of service, better outcomes and better cost efficiency.^1^, ^2^, ^3^, ^4^ Integrated care has been defined as ‘an approach to strengthen people‐centered health systems through the promotion of the comprehensive delivery of quality services across the life‐course, designed according to the multidimensional needs of the population and the individual and delivered by a coordinated multidisciplinary team of providers working across settings and levels of care’.^5^ This holistic personalized perspective on clients pays attention to the origin of clients' symptoms on a psychological, mental, medical and (psycho)social level and consciously adopts their needs, preferences and perspectives.^6^ The advantages of integrated care apply especially to clients with multiple problems (CWMPs), as they need different services from different social support and care providers at the same time to address all their needs.^7^, ^8^ CWMPs are people who experience various combinations of mental illness, intellectual disability, acquired brain injury, physical disability, physical conditions, behavioural difficulties, homelessness, social isolation, family dysfunction and addiction.^8^

Integrated care has been studied extensively. Nevertheless, despite numerous studies, the evidence that integrated care leads to improved outcomes is dispersed and inconsistent.^4^, ^6^ Integrated care is most often studied as a phenomenon taking place at the system, organizational, professional and clinical levels, including functional and normative dimensions.^9^ Many studies have focused on the barriers, difficulties and effects of cross‐sectoral, cross‐organizational and interprofessional collaboration.^4^, ^7^ With the main focus on these levels of integration, clients often seem to be implicitly conceptualized as passive recipients of care, not as active coproducers of services.^4^, ^10^ Consequently, clients' impact on the establishment and outcomes of integrated care may be overlooked.^10^

In recent years, there is an increasing call in the literature on integrated care for stimulating coproduction. Coproduction in this context is described somewhat ‘idealistic’ as ‘engaging clients, their families and communities in the design, implementation and improvement of services through partnership in collaboration with professionals and providers’.^11^ Active involvement of clients, their families and the community is in this type of literature regarded as an essential condition for the success of integrated care.^12^, ^13^, ^14^ Coproduction or actively engaging clients, families and communities are seen as a valuable route to harness their power, attune services to their needs and increase their ability to self‐care (especially for unserved populations and marginalized groups).^11^, ^14^

While coproduction is seen in the literature on integrated care as something to strive for, in service management literature coproduction is regarded as inevitable and intrinsic to any service experience.^15^, ^16^, ^17^, ^18^ Services have four distinctive characteristics: intangibility (services are intangible before delivery), inseparability (the production and consumption occur during the interaction between professional and client), variability (the service's quality and outcomes are shaped within the interaction between professional and client) and perishability (services cannot be stored).^15^, ^18^ In this body of literature, it is underpinned that services do not have any intrinsic value to their users in advance of their usage. Service organizations can only ‘promise’ a certain experience, but their actual performance is coproduced in the interaction with their users.^15^, ^18^ In that sense, the delivery of integrated care services is always a coproduction, although the level of involvement of both (in)formal caretakers and clients may vary.

To add to the literature on integrated care, we focus on how CWMPs, informal caretakers and formal caretakers coproduce integrated care. In this study, informal caretakers are people who provide unpaid care to the CWMP with whom they have a social relationship, such as a spouse, parent, child, other relatives, neighbour, friend or other nonkin. This informal care involves, for example, help with household chores or other practical errands, transport to doctors or social visits, social companionship, emotional guidance or help with arranging professional care.^19^ In accordance with the service management literature, we consider integrated care as inevitably coproduced, although the level of involvement of the participants may vary. Our main question is as follows: How do CWMPs and (in)formal caretakers coproduce integrated care and support? We use data gathered among CWMPs and their (in)formal caretakers in Rotterdam, the Netherlands.

## 2 METHODS

We chose a qualitative research design for this study because the coproduction of integrated care is a complex and multidimensional phenomenon, which is hardly studied. Qualitative methods help us provide rich descriptions of this phenomenon and will help enhance our understanding of the context as well as the underlying mechanisms.^20^

### 2.1 Setting

Data were collected among ambulatory CWMPs. CWMPs are an interesting group of clients to study how integrated care is coproduced. It is widely acknowledged that people who have problems on psychological, mental, medical and (psycho)social levels need a continuum of care designed according to their multidimensional needs delivered by different actors, services and facilities involved on multiple levels of welfare, health care and social services to address all their needs.^5^, ^21^

Data were collected in five districts in Rotterdam, the Netherlands: Bloemhof, Hillesluis, Lage Land, Ommoord and Lombardijen. Rotterdam is the second largest city in the Netherlands and is known for its large population of people with socioeconomic and (psycho)social problems. In the selected districts, large concentrations of these people can be found, although Ommoord scores slightly better compared to the other four districts.^22^

Since 2015, as part of a major welfare state reform in the Netherlands, responsibility for social care and support, basic income provisions and youth care have been decentralized from the central government to municipalities. The idea behind this decentralization is that municipalities are more capable than the national government of being responsive to local needs and can provide tailored, integrated care as they are (literally) closer to clients. The reform was envisioned as a transition from a welfare state to a participation society, which places greater emphasis on citizens' individual responsibility, engaging civil society and shrinking the role of the state.^23^, ^24^ Traditional roles (citizen as client) were reshaped (citizen as coproducer).^25^

### 2.2 Participants

CWMPs were recruited via community‐based primary care teams (CT) professionals (CPs). As part of the implementation of the welfare state reform, a community‐based primary CT was established in every district in Rotterdam. Community‐based primary CPs are assigned by the municipality of Rotterdam to completely assess the multidimensional needs of CWMPs and organize integrated care. Citizens can only turn to CPs when they are faced with multiple problems. CPs have different disciplinary backgrounds, for example, social psychiatric nurses, youth care workers, social workers, community workers, counsellors for elderly individuals and intercultural workers. The procedures prescribe that CPs map out the CWMP's multidimensional needs within the first 6 weeks. Based on this assessment, the CPs, together with CWMPs and their informal network (if available), are expected to organize integrated care. CPs provide support themselves and work together with professionals in their teams and with professionals across the boundaries of their teams, such as housing corporations, general practitioners, addiction therapists, mental health organizations, charity and religious organizations and CWMPs' informal networks. CPs have 6–9 months to organize care and support and refer the CWMPs to the appropriate professionals and institutions for follow‐up, if necessary.

Our aim was to follow CWPMs for 1 year, from the start of their involvement with a CT, until several months after a referral from the CT. This allowed us not only to track and reconstruct the entire coproduction process but also to see the longer‐term effects. CPs were asked to inform CWMPs within the first 6 weeks of their involvement with CWMPs. A period of 6 weeks was chosen in coordination with CPs. CPs indicated that 6 weeks were needed to introduce the study properly, for example, to establish an initial trust relationship. As inclusion was difficult at this study's start, an incentive (a 10‐euro gift card) was introduced. Incentives increase the likelihood of participation but could negatively affect the data collection or the human subject.^26^, ^27^ We, however, think that the conditions that may lead to a negative impact were absent in our study: subjects were not in a dependent relationship with the researcher, the study is not degrading and the incentive was not that high that it would overrule participants possible aversions.^27^

**Figure 1:**
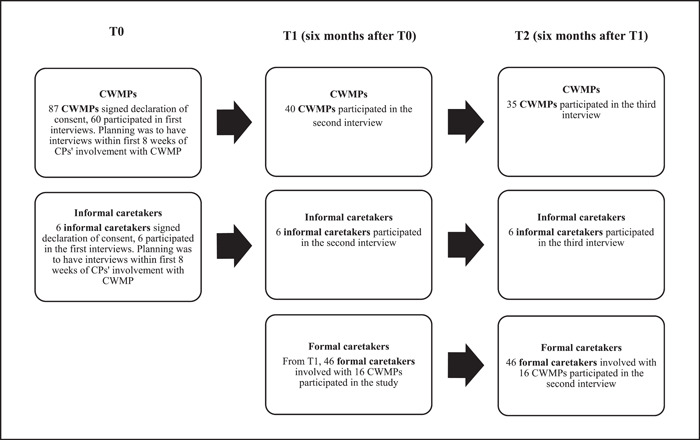
Data collection process

All CWMPs signed a declaration of consent before participation. CPs ensured that CWMPs understood the study's content via an extensive oral explanation. Figure 1 gives an overview of the data collection process.

Due to different types of circumstances, such as imprisonment, mental breakdown, stress overload of the participating CWMPs and struggles to contact them (e.g., disconnected phones or CWMPs not answering their phone), our interview planning was not always attainable. This challenge is inherent to CWMPs' situation and characteristics.^26^ Most interviews were held around the scheduled date, with a maximum deviation of 3 months. The clients' characteristics and reasons for nonparticipation in T0, T1 and T2 can be found in Appendix A.

(In)formal caretakers were recruited via participating CWMPs. All CWMPs were asked whether the researchers could interview their informal caretakers at T0. Although we aimed to also include informal caretakers in our study, the reality was that many CWMPs did not have informal caretakers (e.g., they lost their informal network as their problems increased), did not want to involve their informal network in the care trajectory or they did not have an informal network that could contribute to the care trajectory (e.g., informal network occupied with their own (multiple) problems or consisted of criminals or addicts).

At T1, via a purposeful sampling strategy, 16 CWMPs were asked whether all involved formal caretakers could be interviewed. Cases varied, such as excellent or rich examples of cases, cases representing a variety of typical situations and cases meeting predetermined criteria (e.g., CWMP).^28^, ^29^ The inclusion of involved formal caretakers at T1 was decided after experiences with their inclusion at T0. A trust relationship was necessary for CWMPs to feel confident that it would not harm their support trajectory or privacy. Forty‐six formal caretakers participated in this study. The (in)formal caretakers' characteristics, including reasons for nonparticipation, can be found in Appendix B.

Data in the municipal record systems about participating CWMPs were also examined. In this system, CPs and other professionals working for the municipality recorded all interactions with CWMPs, informal caretakers and other professionals, CWMPs' support plan and assessment of their self‐reliance. Alongside data collected via interviews, data from the municipal record system helped to get an overview of the timing, frequency and nature of interactions among CWMPs, informal caretakers and other professionals. It also provided information on what professionals recorded after (re)assessing the CWMPs' situation with the CWMPs including (revised) plans and actions to deal with the CWMPs' situation during the care trajectory. This data was used to substantiate the data collected via interviews and (re)construct the coproduction process of integrated care during the care trajectory (including a timeline). The four sources of data (interviews with CWMPs, informal caretakers, formal caretakers and data from the municipal record system) collected over multiple time points allowed us to gain insight into the process of coproducing integrated care, including all participants' considerations, perceptions and evaluations during this process.

### 2.3 Interviews

Data were collected between September 2015 and March 2018 using a semistructured interview guide. The central themes in all interviews were the interviewee's understanding of the CWMPs' situation and problems, their perspective on the CWMP's support needs, their evaluation of the quality and adequacy of care provided, their (evaluation of their) role and those of others involved in the support trajectory, the level to which integrated care was provided and their perspective on CWMPs' future. Formal caretakers were also asked about the circumstances under which they can provide CWMPs the care needed, their interaction with other (in)formal caretakers and their knowledge about the care provided by other (in)formal caretakers. Each theme relates to aspects of integrated care and coproduction. Especially, the themes that focus on the interviewee's understanding of their role and those of others involved in the support trajectory, formal caretakers' interaction with other (in)formal caretakers and their knowledge about the care provided by other (in)formal caretakers used to delve deeper into the coproduction aspect of integrated care.

Interviews with CWMPs and informal caretakers lasted between 45 min and 2 h, and those with formal caretakers lasted between 30 min and 1 h. All interviews were audiotaped and transcribed verbatim.

### 2.4 Data analysis

Data were pooled and analysed by two authors (LR‐dB and JvW) using Luborsky's^30^ technique for thematic analyses. This process includes becoming acquainted with the data by reading the texts, the development of preliminary themes (open coding), axial coding and selective coding. At each step, the data and developed themes were discussed among the two authors, and an intercoder agreement was reached. The data analysis followed a deductive and inductive analysis process. Deductive in the sense that we, for example, analysed in each care trajectory how the CWMP's multidimensional needs were assessed, how care was designed and implemented according to these needs (aspects of integrated care) and how this process was the result of active involvement or engagement of CWMPs and their (in)formal caretakers (coproduction). Inductive in the sense that new themes and codes were created through the analytical process. Themes that were inductively developed related among others to ‘crisis, stress, complexity, trust, reflexivity and iterative’. Based on these themes two types of cases were identified in which professionals dealt differently with these issues and clients were involved differently. Data were analysed using Atlas.ti.

### 2.5 Ethics

The ethics review board confirmed that our study was outside the scope of the Netherlands' Medical Research Involving Human Subjects Act and that the rights and privacy of study participants were sufficiently considered (MEC‐2017–348). All participants signed a declaration of consent and could withdraw from the study at any moment for any reason. One CWMP withdrew from the study during an interview due to emotional instability; other reasons for withdrawing can be found in Appendix A.

## 3 RESULTS

To outline our findings, we follow the timeline of our cases. Our involvement starts when CWMPs reach out for help from the municipality (start care trajectory) and stops after 1–1.5 years. The start of the care trajectory is a relative concept in this context. Most CWMPs have been involved with many (public) services and care trajectories, often from early childhood, before we start to follow them. Therefore, some CWMPs reach out for help from the municipality while actively following another care trajectory, and not all care trajectories are completed when our involvement stops. Following the timelines of our cases, we first outline how the client's multidimensional needs are mapped out. We then outline two distinct types of cases in which various levels of integrated care are coproduced.

### 3.1 Assessing multidimensional needs

Our data indicate that CWMPs' multidimensional needs are rarely completely assessed at the start of care trajectories. We found several reasons for this.

#### 3.1.1 The crisis first

Most CWMPs enter the support trajectory with massive problems, mostly acute needs, which require immediate action to avoid further escalation. For example, CWMPs are confronted with pending house evictions, have had their utilities turned off, have escalating debts, are homeless, have no income, have no health insurance, have no ID or are heavily addicted. CWMPs feel highly anxious and want their urgent problems to be solved and have their stress level reduced. Consequently, CWMPs' initial problem description focuses on their urgent problems in which they emphasize the need to have these issues resolved.

I had so many problems, so many problems, also debts. I had to write letters … couldn't do it myself. (…) I have a wife, a baby on the way, those financial problems made me crazy and had to be solved. (C36)

Additionally, many CPs (and other professionals) believe that the multidimensionality of CWMPs' situations can only be truly assessed when their urgent problems are addressed and their stress level has decreased.

My first focus was to calm things down. Her financial problems caused a lot of stress and increased her physical problems. (…). She [C23] had no insurance, and her utilities were going to be turned off. These are such basic needs. Those matters had priority. The other things would take more time [other underlying problems, such as her mental health]. It was not immediately made an important topic. (Community‐based primary care team worker C23a)

Some formal caretakers also notice that CWMPs attract formal caretakers with a hands‐on mentality who enjoy managing crises, causing them to overlook the multidimensionality of CWMPs' situations.

I think that we as caretakers overlook things [already involved caretakers or problems] because we dive into problems too quickly and get to work. We are often dealing with crises that cause us to BAM!, start acting. Then, halfway through, we find out all types of things [problems, involved people, interventions that do not work out]. That's a shame (…) We want to help. (…) I like crises. There must be pressure. (Community‐based primary care team worker C23b)

#### 3.1.2 Partnership is built on trust

Another complicating factor for assessing CWMPs' multidimensional needs is the lack of trust among CPs (and other professionals) and CWMPs at the start of the care trajectory. Almost all CWMPs in this study have a deep‐rooted distrust of public service providers or ‘the government’, mostly due to negative experiences with the public service system in the past. Their distrust prevents them from sharing information beyond the (urgent) problems they want to be helped with.

In my situation, it's all caused by the municipality [of Rotterdam]. Because of the municipality, I ended up having rent arrears. Social services gave me too little money [income earned months before was deducted from his social benefits]; if I get too little money, I cannot pay my rent. It is called ‘social service’ and not ‘social misery services’. (C54)

Therefore, most CWMPs are reluctant to share information about, for example, things they are ashamed of, illegal activities they are involved with or more private matters. This withheld information can be potentially relevant information for assessing CWMPs' multidimensional needs.

C80 enters the support trajectory with massive debts. She says that after she ended her beauty salon, her accountant appeared to have never paid taxes. C80's community‐based primary care team worker starts to help C80 with her debts. After a couple of months, C80's community‐based primary care team worker finds out that C80's debts are not caused by her accountant but by C80's criminal activities and related conflicts.

Many formal caretakers are aware of the importance of a good relationship with CWMPs. At the start, for many of them safeguarding the relationship outweighs the importance of obtaining insight into CWMPs' multidimensional needs. When CWMPs are reluctant to share information, many professionals respect this.

#### 3.1.3 A veil of complexity

The complexity of CWMPs' problems also hinders the understanding of CWMPs' multidimensional needs.

C23 has had problems in several areas of her life. She used to have a cocaine addiction, had bladder cancer, had several abusive relationships, went through several traumatic events, had Gilles de la Tourette, and had major financial problems (e.g., threats to shut off her utilities).

As in C23's case, CWMPs deal with problems in many areas of their lives. What makes it difficult to see through the (veil of) complexity of these problems is that they often have a great number of problems (e.g., it is difficult to map out all problems), CWMPs' problems are intertwined (e.g., making it challenging to unravel them) and it is difficult to understand how these problems affect daily life and current problems. Additionally, CWMPs' attitudes towards potential underlying problems vary. Many CWMPs do not want to explore the multidimensionality of their problems. For example, they ignore the layeredness of their problems, lack insight into their disease or are afraid of diving deeper into the origins of their problems (e.g., afraid of mental instability and traumas). Others are more open to exploring their underlying problems but, together with formal caretakers, struggle to see through this complex puzzle.

### 3.2 The crisis is not curbed quickly

Our data show that all care trajectories start with addressing the urgent problems first but also show that this ‘crisis phase’ is often of long duration (several months to a year). Solving urgent problems usually implies going through several interdependent (bureaucratic) procedures, such as the application for social benefits, a municipal postal address and debt rescheduling. These bureaucratic procedures use predefined steps with limited forgiveness for CWMPs' mistakes or deviant behaviour. CWMPs struggle to successfully complete these processes, and formal caretakers must invest a great deal of time to help CWMPs with this.

[C56] had no money at all, nothing. The woman would not accept our help if it cost her money [support would cost her health insurance excess]. We arranged funds to pay for this for her. We left her psychiatric situation for what it was, until the basics were rearranged [woman has schizophrenia] (…) We have arranged special administration, reconnected her utilities [utilities were turned off]. Her finances are now arranged. (…) Before you can write to all money claimants, special administration must be arranged, many steps must be taken. [We must] collect all necessary documents, bank account statements, make copies of these documents, etc. She also needed to be seen by an independent psychiatrist [for the application of special administration]. Then, it is up to the court, which takes a few weeks before the judge decides. (…) This is a process of months, not something done in a couple of weeks. (Psychiatric nurse C56)

In only two cases in this study were the most urgent problems of CWMPs relatively quickly solved, creating room to further analyse the multidimensionality of these CWMPs' situations.

In sum, our data indicate that CWMPs' multidimensional needs are rarely completely assessed at the start of CWMPs' care trajectories. Additionally, starting from the client's perspective does not automatically lead to an integrated approach.

### 3.3 The coproduction of integrated care

Nevertheless, our findings show that despite the absence of a full understanding of CWMPs' multidimensional needs at the start and reluctant clients, integrated care can be achieved. We found two types of cases in which different levels of understanding of CWMPs' multidimensional needs and integrated care were finally established. Table 1 gives an overview of the key elements of the two types of cases.

**Table 1:** Overview of key elements case types 1 and 2

Case type 1	Case type 2
CWMPs' multidimensional needs are not completely assessed at the start of the care trajectory.	CWMPs' multidimensional needs are not completely assessed at the start of the care trajectory.
Both CWMPs' and formal caretakers' actions are aimed at addressing urgent problems first. CWMP's multidimensional needs are ignored until urgent problems are solved.	From the start, formal caretakers take initiative to not only address the CWMP's urgent problems, but also to explore the multidimensionality of CWMP's needs together with other formal caretakers.
Solving urgent problems takes more time than anticipated beforehand due to CWMP's underlying problems in combination with the complexity of bureaucratic procedures.	Experiences gained during the first period, in which both urgent problems are addressed, and the multidimensionality of CWMPs' needs is explored, are used to revise involved actors understanding of CWMPs' multidimensional needs and tailor interventions.
The care trajectory's progress and approach are reconsidered by both formal caretakers and CWMPs. At this moment in time, many CWMPs get disappointed, lose motivation and even leave the care trajectory. Formal caretakers take more initiative to redirect the course of the care trajectory. Collaboration with other formal caretakers is intensified and formal caretakers try to redirect the client to get the urgent problems solved. Focus remains on solving urgent problems first, and multidimensionality of CWMPs' needs are not explored (yet).	Urgent problems are often more quickly addressed than in type 1 cases.
Finally, formal caretakers and CWMPs manage to solve the urgent problems, yet this takes more time than anticipated. Underlying problems are usually not addressed, and CWMPs are still very vulnerable. This vulnerability makes them susceptible to new crises. Several relapse into similar problems within the 1–1.5 years we followed these CWMPs.	In successful type 2 cases, CWMPs seem to leave the care trajectory less vulnerable than in type 1 cases. CWMPs have more often gained (some) insight into the multidimensionality of their situation and have a more positive image about public services.

Abbreviation: CWMPs, clients with multiple problems.

#### 3.3.1 Case type 1: Solutions to problems

C60 is addicted to heroin, has war traumas, is homeless, has no income, struggles with feelings of loss, and stays in a religious community. C60 wants a normal life. C60's community‐based primary care team worker starts to help C60 regain his necessities. She concludes that he needs a postal address to be able to apply for social benefits and social housing. She also notes his war traumas and addiction.

Case type 1 cases represent most cases in our study (80% of the cases). In these cases, at the start, solving urgent problems is the sole focus of CWMPs and formal caretakers (‘solutions to problems focus’). In C60's case, this implies getting him a postal address so he can apply for social benefits. In case type 1, the multidimensionality of CWMPs' situation is ignored until the urgent problems are solved. The care trajectory is approached as a linear process (urgent problems first, then diving deeper into the multidimensionality of CWMP's situation).

As multidimensionality is ignored, the help CWMPs receive and the interactions among CWMPs and formal caretakers have a practical focus, for example, how the CWMP can apply for social benefits, what documents need to be collected and how to best interact with formal bodies (e.g., creditors or social services). During interactions, formal caretakers and CWMPs mostly exchange practical information. The same applies to interactions among formal caretakers. Formal caretakers most often exchange information about what has been and still needs to be done to address urgent problems. It also stood out that in type 1 cases, formal caretakers more often tend to work solo.

All formal caretakers involved with C60 have contact with each other about practical matters (who does what, what has been done), except his addiction therapist and people from the religious community. His addiction therapist does not want to be involved (he thinks it is not necessary to do his work). People from the religious community are not considered relevant for the care trajectory by other formal caretakers.

However, this often changes when it becomes clear that urgent problems are more difficult to solve than expected.

From the start, C60 does not keep appointments with any formal caretaker involved. He also struggles to collect the documents necessary to apply for social benefits. C60's behaviour delays the application for social benefits. C60's challenges with engaging in the care trajectory leads the involved formal caretakers to wonder why.

When progress is not being made, formal caretakers start to look beyond the most urgent problems. This triggers the need to align actions with other formal caretakers and go beyond practical matters. Contact among formal caretakers is intensified and starts to become more reflexive; what may be the underlying causes? Interactions between formal caretakers and CWMPs also start to change. However, CWMPs often become disappointed at this point and lose their motivation. Some CWMPs even decide to exit the care process. This attitude is reflected in the way they express themselves to formal caretakers. Formal caretakers start to initiate conversations with CWMPs about why progress is not being made and try to reflect on potential reasons, for example, they confront CWMPs with their (destructive) patterns and own responsibility and try to determine what is hindering CWMPs from moving forward. The initial linear process becomes more iterative and reflexive.

After 6 months, C60's social benefits are granted. His debt counsellor has been replaced. In hindsight, she believes C60 should have received more specialized support. C60's community‐based primary care team worker is not sure what is truly going on with C60, possibly his heroin addiction or brain damage due to his addiction. She continues to encourage C60 to show up to appointments and collect his documents with little success.

During the summer holiday, fewer people are in the religious community, and C60 increases his drug use and lies in bed a lot. He misses more appointments, and involved formal caretakers struggle to contact him. C60's community‐based primary care team worker and the debt counsellor arrange a meeting with C60 to reconfirm their agreements. C60 says it is chaotic in his head, and he feels overburdened.

However, this reflexivity continues to have a practical focus, namely, on what needs to be altered to solve the urgent problems (still a solution to problems focus). In C60's case, the focus on arranging his social benefits continues. C60 is encouraged to show up at meetings, answer his phone and put effort into collecting his documents. Formal caretakers and C60 do not reflect upon his increased drug use (this is even ignored). An in‐depth or comprehensive understanding of the multidimensionality of the CWMP's situation is usually not gained.

In type 1 cases, formal caretakers and CWMPs manage to solve the crisis, yet this takes more time than anticipated beforehand. Underlying problems are usually not addressed, and CWMPs are still very vulnerable. This vulnerability makes them susceptible to new crises. Even during the time in which we followed CWMPs, we saw several of them relapse into similar problems, as occurred with C60. The crisis often leaves lasting marks: making CWMPs feel less competent to deal with challenges in life and less in control.

After 1 year and after a period of six months of having social benefits, C60's social benefits have ended. He did not comply with appointments made (he left the country and missed several appointments). In hindsight, C60's community‐based primary care team workers believe that he should have received more specialized care, and more attention should have been paid to underlying problems, such as C60's mental welfare. C60's community‐based primary care team worker was not aware of C60's increased heroin use. After 14 months, C60 is referred to an organization specializing in people in recovery and ex‐cons. C60 feels unfairly treated; he has no idea what was expected of him and seems unable to reflect on his own role.

#### 3.3.2 Case type 2: An iterative process

C39 lives on the proceeds of a house he previously sold, is in arrears (eviction pending), has troubled relationships, and has severe health problems (e.g., has approximately 5% vision due to cataracts).

One day, C39's is evicted by the housing association. C39 is surprised. He did not know about the debts (never opened his letterbox). The eviction is averted when C39 accepts C39's community‐based primary care team worker's help.

In type 2 cases (20% of the cases), CWMPs and formal caretakers also start with solving urgent problems.

C39's community‐based primary care team worker starts to immediately deal with C39's urgent problems. She starts to organize his mail and debts, plans an appointment with a trustee, and reaches out to formal caretakers from the housing association. She also reaches out to people in C39's informal network (with C39's consent). Initially, C39 doubts whether this is necessary, but C39's community‐based primary care team worker convinces him it is.

In this case, from the start and alongside interventions to address urgent problems, formal caretakers take the initiative to come to a shared insight into the multidimensionality of the CWMP's situation. Formal caretakers take the initiative to contact other involved formal caretakers and people from the CWMP's informal network. They have conversations about practical matters but also initiate discussions about potential underlying problems and the adequacy of interventions. For example, C39's community‐based primary CT worker reaches out to C39's friends and children. She invites them to share their perspectives on C39's situations and vice versa.

C39's community‐based primary care team worker makes an appointment with C39's GP for his eye problems and feelings of depression. C39's community‐based primary care team worker goes with C39 to his GP and ophthalmologist. She picks him up in her car. C39 appreciates this a lot. When C39 is truly short of breath, C39's community‐based primary care team worker brings him to the hospital and stays with him until the treatment is finished in the evening.

C39's community‐based primary care team worker is compassionate but also direct and confrontational. For example, she confronts C39 with a potential unhealthy relationship with a woman and her belief that C39 dwells in feelings of grief. C39 appreciates his community‐based primary care team worker's directness and thoroughness.

Formal caretakers also take initiative during interactions with CWMPs to come to a shared understanding of the multidimensionality of their situation. Our study shows that CWMPs mostly consider external reasons as causes for their problems. These formal caretakers also confront them by discussing the CWMPs' own involvement in their problems.

Several interventions are implemented, not all equally successful. For example, the trustee is formally assigned by the court. This is a massive relief for C39. He appreciates he no longer receives mail, and his finances are arranged. Domestic support is arranged to help C39 keep his house clean (C39 is not open to this).

In type 2 cases, solving urgent problems is not a linear process. Although many of these formal caretakers also believe the CWMP's multidimensional needs could only be truly assessed when their urgent problems are addressed, many view this period as helpful to gain more insight into the multidimensionality of CWMPs' situation. Experiences gained during this period are used to continuously revise involved actors' understanding of CWMP's multidimensional needs and tailor interventions (iterative process).

C39's ex‐wife dies. He is shattered by the news. C39 gets into another conflict with his GP. His debts are solved, although with some hiccups. C39's eye problems are solved with surgery. C39's community‐based primary care team worker ends her support. In hindsight, C39's community‐based primary care team worker hoped to address more of C39's problems, but he was not open to this. For example, his inguinal hernia, his teeth, and potential mental problems which caused him to get in trouble. During the interviews, C39 shares that he knows he could have more help, and C39's community‐based primary care team worker thinks he should address more problems, but he does things at his own pace. When needed, he will reach out for help again.

In type 2 cases, multidimensional needs are often more completely assessed than in type 1 cases. However, formal caretakers can only encourage CWMPs to address their needs, and CWMPs ultimately decide on what needs are addressed. If CWMPs do not want to address certain needs, formal caretakers cannot force them to do so. However, in successful type 2 cases, CWMPs seem to leave the care trajectory less vulnerable than in type 1 cases. CWMPs have more often gained (some) insight into the multidimensionality of their situation and have a more positive image of public services.

## 4 DISCUSSION

In recent years, there is an increasing call in the literature on integrated care for stimulating coproduction. Coproduction in this literature is described as actively engaging clients, families and communities and is seen as a valuable route to harness their power, attune services to their needs and increase their ability to self‐care (especially for unserved populations and marginalized groups).^12^, ^13^, ^14^ It is also part of a fundamental paradigm shift in which people are put at the heart of services and paternalistic care is abandoned.^11^, ^14^ In this study, we show that there is always a level of coproduction required to establish integrated care, especially for CWMPs. Client involvement is indispensable to assess their complex needs, but also during service delivery. However, stimulating a more active role of CWPMs in coproduction does not seem to increase, but may even hinder the delivery of integrated care.

Foremost, our study shows that in practice, the multidimensionality of CWMPs needs, which should function as the organizing principle of integrated care, are often not completely assessed at the start of CWMPs' care trajectories. Important reasons behind this are the urgency of the specific problems with which CWMPs enter the support trajectory, their lack of trust in government institutions and the complexity of their problems. Basically, CWMPs are at the start often unwilling and unable to look beyond their most urgent problem(s). We furthermore identified two types of cases. In both types, we see professionals trying to coproduce integrated care with clients. But only in one case type, do they seem to succeed. In case type 1, formal carers follow the wishes of the CWPM to only focus on the problems they consider urgent. At the start, CWPM and carers have more or less equal roles. However, when progress is not forthcoming, caregivers feel obliged to take the lead and also look at underlying problems (a more paternalistic approach). As the focus remains however on solving urgent problems, this does not result in integrated care. In case type 2, from the start, formal caretakers direct the care trajectory, and in a sense, take the lead. CWMPs' expressed needs (get urgent problems solved) are respected and actions are taken to get these solved. However, from the start, formal caretakers also direct and prepare the process to further analyse the multidimensionality of CWMPs' needs (although this is not what CWMPs ask for) together with other formal caretakers. Later, in the process, they also motivate CWPMs to work on other problems, thereby stimulating the delivery of integrated care and support. These observations raise questions about the extent to which paternalistic care is something to leave behind for this group of unserved and marginalized clients. It seems that to stimulate integrated care for these clients, formal caretakers must take the lead in exploring the multidimensionality of CWMPs' needs and in designing and implementing care according to these needs. Another important finding is that for this client group especially, the coproduction of integrated care cannot be approached as a simple linear process, which starts with a diagnosis (identifying multidimensional needs) and is then followed by the delivery of care and support. Our study indicates that the coproduction of integrated care should be viewed as an iterative process. It is something that needs to be worked towards via iterative steps in which the CWMP's multidimensional needs and interventions are continuously revised, deepened and sharpened.

These conclusions lead to several reflections on the literature on integrated care, the role of formal caretakers, current policies aimed at integrated care and bureaucratic processes.

One of the core principles in integrated care is that clients should be put at the centre and care should be organized in line with clients' multidimensional needs.^9^, ^11^, ^31^, ^32^ These principles are not disputed in this study. We see that when the multidimensionality of CWMPs' situation is not considered an urgent problem are approached in isolation, care trajectories often fail. Most studies on integrated care implicitly conceptualize clients as passive care recipients, while we found that integrated care delivery is very much dependent on the willingness of clients to participate in its coproduction. At the same time, our study shows that involving clients and putting them in the centre does not automatically stimulate an integrated approach. As we have seen, CWMPs do not initiate (and may even hinder) a multidimensional assessment of their situation and are often not expecting (or even wanting) an integrated approach. Formal caretakers seem to have a key role in initiating integrated care for this client group. This approach requires formal caretakers who can build strong trust relationships with CWMPs, can organize shared reflexivity to unravel the complexity of CWMPs' situations, and can take on supportive, compassionate and confrontational roles (coaching). However, even then, there are no guarantees that this will result in integrated care delivery, as not all clients will be enticed to participate in coproducing integrated care.^17^

Furthermore, our study shows that for delivering integrated care, formal caretakers experience difficulties not only because of the fragmented delivery system, as is often discussed in the literature but also because bureaucratic procedures mostly follow a linear logic.^4^, ^7^, ^9^, ^32^, ^33^, ^34^, ^35^ These procedures stipulate that in predefined steps, starting with a multidimensional diagnosis, CWMPs and formal caretakers (must) work towards an outcome (e.g., social benefits or debt restructuring). While these procedures safeguard equal treatment of equal cases, they do not facilitate or initiate iterative processes. Consequently, formal caretakers must invest a considerable amount of time, in bringing together the fickle processes of helping CWMPs go through these linear bureaucratic processes. The bureaucratic process also steers formal caretakers towards a linear instead of an iterative process. This could be an important insight for policymakers in the Netherlands and other European countries who implement policies aimed at integrated care.^36^, ^37^, ^38^

### 4.1 Limitations

In this study, we focused on a specific population, that is, CWMPs in Rotterdam, the Netherlands. Nevertheless, the specific policy context emphasizing integrated care and Rotterdam provided an interesting setting, as vast numbers of CWMPs can be found in this city, especially in the districts we focused on. We acknowledge that the specific population and setting could have affected our results. Therefore, studies on the coproduction of integrated care with other populations and in other settings could help to gain more insight into how integrated care is coproduced at a micro level. We must also acknowledge that the inclusion of people with multiple problems had its challenges. We have conducted our research in a scientifically sound manner, but we had to deal with obstacles in obtaining access to CWMPs and keeping them on board. Including clients via CTs could have created a selection bias. Knowing that CWMPs are difficult to include in research and that our study is one of a few longitudinal studies on CWMPs, we are confident that our study provides interesting insights and can stimulate more research into the care trajectories of these types of complex clients.^39^, ^40^ Another limitation of this study is that we struggled to include CWMPs' informal caretakers. Although we tried, we were only able to include a few informal caretakers. We therefore could not reflect on the role of informal caretakers in the coproduction of integrated care.

## 5 CONCLUSION

Our study shows that integrated care does not come naturally when CWMPs are put at the centre and that formal caretakers have a key role in initiating integrated care. The linearity of many bureaucratic processes does not enhance and even hinders the establishment of integrated care. Based on this study, we also conclude that clients should be considered active actors in every study on integrated care.

## CONFLICT OF INTEREST

The authors declare no conflict of interest.

## Data Availability

The data that support the findings of this study are not available due to privacy or ethical restrictions. The data are not available on request due to privacy and ethical restrictions.

## APPENDIX A

See Table  A1.

**Table A1:** Participants' characteristics

	T0 all participants who signed declaration of consent	T0 participants who participated in first interview	T1	T2
Sex				
Male	44	32	23	22
Female	43	32	17	13
Total	87	64	40	35

	T0		T1	T2
Age (years)				
25–50	26		9	9
50‐75	33		26	21
75–100	5 (oldest 86 years)		5	5
Total	64		40	35
Living circumstances				
Alone	28		24	21
Alone/without a partner or roommates and with children	9		2	2
With partner/roommates	6		4	4
With partner/roommates and child(ren)	13		8	7
Homeless	6		1	1
Homeless with child(ren)	2		1	0
Total	64		40	35
District				
Bloemhof	21		12	11
Hillesluis	7		3	3
Lage Land	8		7	5
Lombardijen	10		6	4
Ommoord	18		12	12
Total	64		40	35
Type of problems^a^				
Finances (e.g., no income or debts)	59			
Daytime activities (e.g., no daytime activities)	30			
Housing (e.g., impending house eviction, homelessness, or contaminated house)	21			
Domestic relationships (e.g., domestic violence or parenting problems)	11			
Physical health	25			
Mental health (e.g., mental problems or mental illness)	36			
Addiction	10			
Activities of daily living	18			
Social network (e.g., absence of a social network or a destructive social network	26			
Participation in society (e.g., no job or no volunteer work)	29			
Encounters with law enforcement system (e.g., [pending] lawsuits for criminal activities)	5			
Nonparticipants: reasons for nonparticipation				
Reason for nonparticipation				
Unreachable	9		12	1
Change of mind, no longer willing to participate	6		9	2
No show	7		3	2

^a^ We used data gathered by the primary care teams complemented with the data from the interviews to provide an overview of the problems the participants in our study faced. We categorized the problems in line with the tool the primary care teams used to identify problems: the self‐reliance matrix (in Dutch: de zelfredzaamheidsmatrix). This tool helps to identify problems in different life domains. All the participants had problems in different life domains.

## APPENDIX B

See Table B1.

**Table B1:** Formal and informal caretakers' characteristics

Type of professional	Organization	Number
Informal caretakers	N/A	6
Community‐based primary care team professional	Municipality of Rotterdam	20
Social worker	Organization for addiction treatment Organization for people with acquired brain injury Religious social work organization Organization for sheltered living	7
Psychiatric nurse	Mental health organization Organization for addiction treatment	2
Psychiatrist	Mental health organization	1
Trustee	Trustee's office	6
Debt counselor	Organization for forensic and specialized care Voluntary organization for debt counselling Debt counselling organization	3
Spiritual caretaker	Organization for spiritual care	1
Pro bono legal counsellors	Municipality of Rotterdam	1
General Practitioner	General practice	2
General‐practice‐based nurse specialist specialized in mental health	General practice	1
Social support act professionals responsible for assigning care for which an indication from the municipality was necessary (in Dutch: Wmo‐consulenten)	Municipality of Rotterdam	2
